# Estimating the Concentration and Biodegradability of Organic Matter in 22 Wastewater Treatment Plants Using Fluorescence Excitation Emission Matrices and Parallel Factor Analysis

**DOI:** 10.3390/s140101771

**Published:** 2014-01-20

**Authors:** Liyang Yang, Hyun-Sang Shin, Jin Hur

**Affiliations:** 1 Department of Environment & Energy, Sejong University, 98 Gunja-dong, Gwangjin-ku, Seoul 143-747, Korea; E-Mail: yangliyang2002@163.com; 2 Department of Environmental Engineering, Seoul National University of Science and Technology, Seoul 139-743, Korea; E-Mail: hyuns@seoultech.ac.kr

**Keywords:** fluorescence spectroscopy, parallel factor analysis, water quality monitoring, wastewater treatment plant, multiple regression analysis

## Abstract

This study aimed at monitoring the changes of fluorescent components in wastewater samples from 22 Korean biological wastewater treatment plants and exploring their prediction capabilities for total organic carbon (TOC), dissolved organic carbon (DOC), biochemical oxygen demand (BOD), chemical oxygen demand (COD), and the biodegradability of the wastewater using an optical sensing technique based on fluorescence excitation emission matrices and parallel factor analysis (EEM-PARAFAC). Three fluorescent components were identified from the samples by using EEM-PARAFAC, including protein-like (C1), fulvic-like (C2) and humic-like (C3) components. C1 showed the highest removal efficiencies for all the treatment types investigated here (69% ± 26%–81% ± 8%), followed by C2 (37% ± 27%–65% ± 35%), while humic-like component (*i.e.*, C3) tended to be accumulated during the biological treatment processes. The percentage of C1 in total fluorescence (%C1) decreased from 54% ± 8% in the influents to 28% ± 8% in the effluents, while those of C2 and C3 (%C2 and %C3) increased from 43% ± 6% to 62% ± 9% and from 3% ± 7% to 10% ± 8%, respectively. The concentrations of TOC, DOC, BOD, and COD were the most correlated with the fluorescence intensity (*F*_max_) of C1 (*r* = 0.790–0.817), as compared with the other two fluorescent components. The prediction capability of C1 for TOC, BOD, and COD were improved by using multiple regression based on *F*_max_ of C1 and suspended solids (SS) (*r* = 0.856–0.865), both of which can be easily monitored *in situ*. The biodegradability of organic matter in BOD/COD were significantly correlated with each PARAFAC component and their combinations (*r* = −0.598–0.613, *p* < 0.001), with the highest correlation coefficient shown for %C1. The estimation capability was further enhanced by using multiple regressions based on %C1, %C2 and C3/C2 (*r* = −0.691).

## 1. Introduction

The lack of clean and fresh water is a great challenge for sustainable development worldwide, thus the re-use and/or recycling of industrial or municipal wastewater after treatment are important to satisfy the growing demand for water [1]. Organic matter present in wastewater poses a major challenge for efficient treatment, since it may cause low coagulation efficiency, disinfection byproduct formation, membrane fouling, oxidant demand, and biomass growth [2–4]. Therefore, it is necessary to develop effective monitoring techniques for organic matter concentrations during the treatment process. Furthermore, a rapid, sensitive and real-time monitoring tool can ensure the reliability of wastewater treatment performance [5,6]. The monitoring of organic matter in the effluents from wastewater treatment plants is also very useful to evaluate the influence of effluent discharge on the water quality, the biogeochemical processes and the ecosystem functions of the receiving water [7].

Bulk organic matter is a complex mixture encompassing a variety of organic compounds which have different susceptibilities to coagulation, adsorption, photo-degradation and biodegradation [2,4,8]. Biodegradability is the most critical factor when evaluating the removal performances of many wastewater treatment plants primarily relying on biological processes [9]. The degree of biodegradability is also closely related to biogeochemical and ecological impacts of effluents on the receiving water [7,10].

Traditionally, the concentration of organic matter in wastewater is measured by total organic carbon (TOC), dissolved organic carbon (DOC), biochemical oxygen demand (BOD), and chemical oxygen demand (COD). The BOD/COD ratio has been used as one of the well-adopted surrogates for biodegradability of TOC [10,11]. However, these measurements are time consuming, limiting their applications for rapid online monitoring.

Recently, fluorescence spectroscopy has been suggested as a reliable optical technique for monitoring organic matter in both natural and engineered systems [5,6,12–14]. Fluorescence measurements are rapid and highly sensitive, need no reagents and require minimum sample pretreatment processes [5]. Thus, they are quite suitable for real-time monitoring. Several *in-situ* fluorometers have been available for continuous monitoring, although the scattering effects of particles need to be minimized using in-line filtering packages when the level of suspended sediments is high [6,15,16]. In particular when fluorescence excitation emission matrices are combined with parallel factor analysis, an advanced data treatment technique (EEM-PARAFAC), it becomes even more effective in identifying individual fluorescent components and tracing their dynamics [16–21]. However, the majority of prior PARAFAC studies have focused on natural environments, while they are rarely applied to engineered systems with the purpose of organic matter monitoring [4]. In addition, the information on the changes of fluorescent components in wastewater treatment plants is limited, and the applicability of EEM-PARAFAC for tracing the concentration, the chemical composition, and the reactivity of organic matter in wastewater treatments is largely unknown. While many of previous studies have focused on a single water treatment plant [20,22], a comprehensive study comparing different types of wastewater treatment plants is much needed.

Therefore, this study aimed to: (1) investigate the changes in the fluorescence intensities and the relative percentages of different fluorescent components in 22 Korean wastewater treatment plants, which are further categorized into five treatment types, and (2) assess the prediction capability of EEM-PARAFAC technique for the conventional organic matter parameters (TOC, DOC, BOD and COD) and the biodegradability (BOD/COD) of the wastewater samples.

## 2. Experimental Section

### 2.1. Sample Collection and Preservation

Wastewater samples were collected in 2 L sterile polyethylene bottles, which were pre-cleaned in distilled water, from 22 different Korean wastewater treatment plants in 2013. Detailed information about the wastewater treatment plants is provided in the supplementary file (Table S1 and Figure S1). The selected wastewater treatment plants have treatment capacities of more than 500 tons per day, and the plants are equipped with separate phosphorous removal facilities, which are based on a variety of physicochemical processes, for the effluent from the biological processes. The wastewater treatment plants in this study were grouped into five categories based on their biological treatment types: activated sludge (AS), biofilm (Media), sequencing batch reactor (SBR), anaerobic/anoxic/oxic (A2O), and membrane bioreactor (MBR). Three types of sewages samples were collected from different locations: the influent before grit chambers, the effluent after the biological treatment processes, and the final treated sewage after the phosphorus removal processes. Samples were kept refrigerated immediately upon return from the field before being analyzed in the laboratory.

### 2.2. Analytical Methods

All analyses were made within one week after the sample collection except for BOD, which was measured immediately after the return from the field. The collected samples were first filtered through a 0.1 mm mesh sieve to remove large sized suspended solids (SS). The levels of BOD, COD, TN, and SS were determined according to the corresponding standard methods [23]. An aliquot of the samples was passed through a pre-ashed GF/F filter and acidified with 1 M HCl to pH 3.0 for the measurements of DOC and fluorescence EEM. DOC concentrations were determined by a TOC analyzer (TOC-V_CPH_, Shimadzu, Tokyo, Japan), with relative precisions of <3% based on repeated measurements. Particulate organic carbon (POC) concentrations were measured on solids retained by the GF/F filters using a CHN elemental analyzer (Flash EA1112, Thermo Finnigan, Waltham, MA, USA). TOC was quantified with the summed concentrations of DOC and POC.

Samples were warmed up to room temperature prior to the fluorescence measurements, following the procedure previously described [16]. Briefly, the samples were diluted until UV absorbance at 254 nm was below 0.05/cm to avoid inner-filter correction [24]. Samples were acidified with 1 M HCl to pH 3.0, to minimize the potentials of metal binding and the subsequent effects on fluorescence [25]. Fluorescence EEM was generated using a luminescence spectrometer (LS-55, Perkin-Elmer, Waltham, MA, USA) by scanning the emission spectra from 280 to 550 nm at 0.5 nm increments, with excitation wavelength from 250 to 500 nm at 5 nm increments. Excitation and emission slits were adjusted to 10 nm and 5 nm, respectively, and the scanning speed was 1,200 nm/min. To limit second order Raleigh scattering, a 290 nm cutoff filter was used for all the samples. The fluorescence response to a blank solution (Milli-Q water) was subtracted from the EEM of each sample [25]. The measured fluorescence intensities were normalized to units of quinine sulfate equivalents (QSE) using the fluorescence of a diluted series of quinine sulfate dehydrate in 0.05 M sulfuric acid at an excitation/emission of 350/450 nm [10,26]. The relative precisions of fluorescence measurements were <2% in triplicate analysis of selected samples.

### 2.3. PARAFAC Modeling

PARAFAC modeling was performed using MATLAB 7.0 (Mathworks, Natick, MA, USA) with DOMFluor toolbox (http://www.models.life.ku.dk) [27]. The number of components was determined based on three diagnostic tools including core consistency, visual inspection of spectral shapes of each component, and split-half validation [27]. The components extracted by PARAFAC represent fluorescence groups that exhibit similar fluorescence properties. The fluorescence intensities of the individual components (*i.e.*, *F*_max_ of C1, C2, and C3), after the dilution factors of the samples were considered, were used as quantity indices of the fluorescence components. The relative contribution of each component to the total fluorescence (*i.e.*, %C1, %C2, and %C3) and the ratios among the components (C2/C1, C3/C1, and C3/C2) were considered as quality indices to represent different chemical composition of fluorescent organic matter.

### 2.4. Statistical Analyses

Correlation analyses, multiple regressions, comparison of means and principle component analysis (PCA) were performed using SPSS Statistics 17.0 software (IBM, Armonk, NY, USA). The correlations in the statistics were evaluated using Pearson and Spearman correlation coefficients (*r* and *ρ*) and the significance levels (*p*). The comparison of means among the five treatment types were tested using the one-way ANOVA. The PCA results were shown in the supplementary file (Figures S2, S3).

## 3. Results and Discussion

### 3.1. Removal Efficiencies of TOC, DOC, BOD, COD and SS in Wastewater Treatment Plants

**Figure 1.**
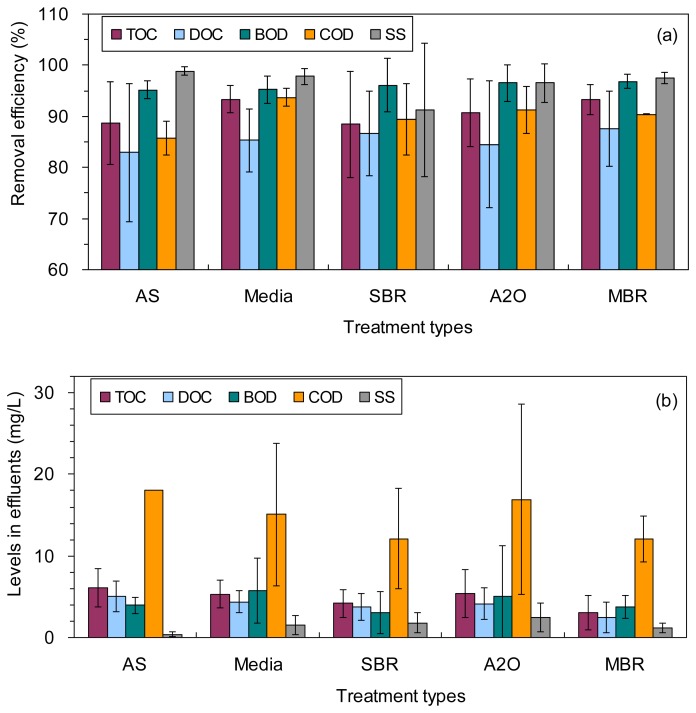
(**a**) Removal efficiencies of TOC, DOC, BOD, COD and SS using different types of wastwater treatments; and (**b**) The levels of TOC, DOC, BOD, COD and SS in the effluents from different types of wastwater treatment plants.

The TOC, DOC, BOD, COD and SS in the influents were 19–142, 17–66, 11–234, 43–467 and 12–283 mg/L, with average values of 74 ± 30, 36 ± 14, 129 ± 59, 208 ± 104 and 88 ± 64 mg/L, respectively (Table 1; Supplementary file Table S2). They were decreased notably to 5.5 ± 2.2, 4.4 ± 1.4, 5.0 ± 5.1, 17 ± 9 and 2.0 ± 1.6 mg/L, respectively, in the effluents (Table 1; Figure 1b). As a result, the corresponding TOC, DOC, BOD, COD and SS removal efficiencies in the wastewater treatment plants are 91% ± 6%, 85% ± 10%, 96% ± 3%, 91% ± 4%, and 96% ± 5%, respectively (Table 1; Figure 1a). Among the four organic matter-related water quality parameters examined, BOD showed the largest reduction from the influent to the final effluent for the wastewater treatment plants investigated (Figure 1a). In contrast, DOC generally showed the lowest removal efficiency for all the wastewater treatments (Figure 1a).

The TOC, DOC, BOD, COD and SS removal efficiencies were not statistically distinguished among the five different treatment types (ANOVA, *p* > 0.05), except for the lower removal efficiency of COD using the AS treatment than using the Media treatment (86% ± 3% *vs.* 94% ± 2%, Table 1; ANOVA, *p* = 0.04). The levels of TOC, DOC, BOD, COD and SS in the effluents showed no significant difference among the five different treatment types (ANOVA, *p* > 0.05; Figure 1b).

### 3.2. Spectral Characteristics of PARAFAC Components

**Figure 2.**
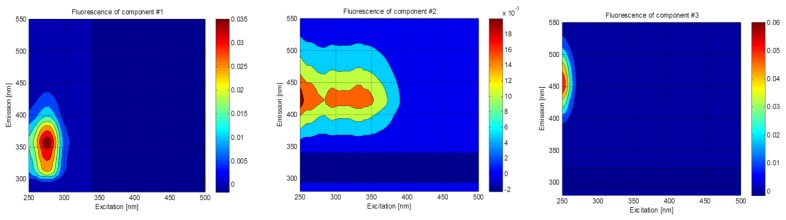
EEM contours of fluorescent components identified using PARAFAC.

Three fluorescent components were identified using PARAFAC in this study (C1, C2, and C3; Figure 2). Component C1 showed a single peak at excitation/emission wavelength of 275/359 nm. The spectral characteristics of C1 were similar to those of tryptophan, which has the excitation/emission maxima of 275–280/340–354 nm [28]. The tryptophan-like component is typically related to the presence of amino acid and/or protein-like substances [16,29] abundant in raw wastewater [8], which can be preferentially removed by microbial degradation [30,31]. Component C2 had three excitation maxima at ≤250 nm, 295 nm, and 330 nm, and one emission maxima at 423 nm. C2 resembled the traditionally-defined peaks A (at 260/380–460 nm) and M (290–310/370–420 nm) [32], and it was also defined as a fulvic-like component [5,33]. It was reported that two similar fluorescence components were removed efficiently in six water recycling treatment plants in Australia [19]. Component C3 showed a strong peak at ≤250/453 nm, which is similar to the peak A, and it was also assigned to humic-like component [4,29]. This component is found in streams and seawater, but it is rarely present in wastewater [18], and little is known about its behavior in the water and wastewater treatment plants based on a recent review paper [4].

### 3.3. Changes of PARAFAC Components in Wastewater Treatments

**Figure 3.**
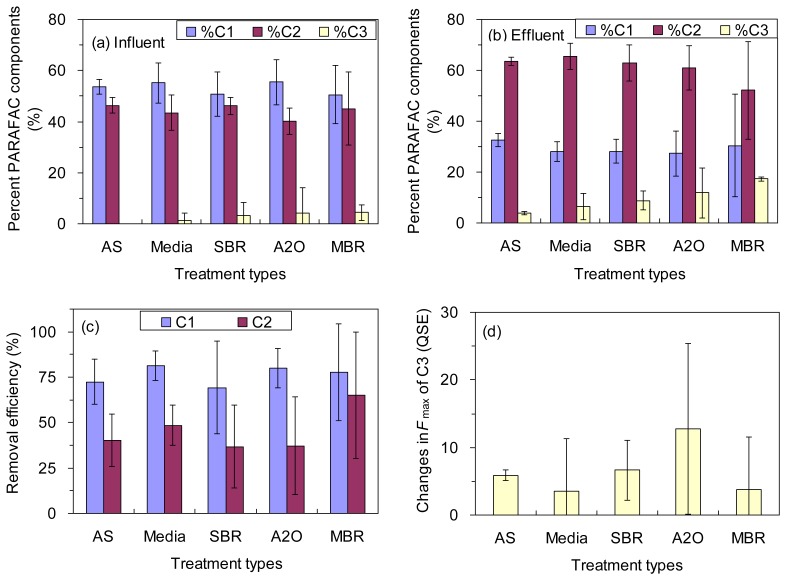
(**a**) The percent PARAFAC components in the influents; (**b**) The percent PARAFAC components in the effluents; (**c**) Removal efficiencies of components C1 and C2; and (**d**) The accumulation of component C3 (an exception of 166 QSE decrease is excluded from the A2O treatment) in different wastewater treatments.

The fluorescence intensities (*F*_max_) of C1, C2 and C3 in the influents ranged from 67 to 335 QSE, from 82 to 240 QSE, and from to 0 to 26 QSE, respectively, with an exception of 231 QSE for C3 in an A2O treatment plant (Table 1). The average values were 208 ± 67, 159 ± 36, and 5.2 ± 9.0 QSE for the *F*_max_ of C1, C2, and C3, respectively. The relative contributions of the PARAFAC components to the total fluorescence (%C1, %C2, and %C3) corresponded to 54% ± 8%, 43% ± 6% and 3% ± 7%, respectively (Figure 3a). Overall, the raw wastewaters were characterized by abundant protein-like fluorescence (C1) and fulvic-like fluorescence (C2), which is consistent with the general features of sewage fluorescence described in previous studies [3,5,12]. In contrast, the level of humic-like fluorescence (C3) was very low in the influents for this study, which were reported in previous studies as well [5,18].

Dramatic changes were observed for the PARAFAC components from the influent to the effluent. The protein-like fluorescence (C1) showed the highest removal efficiency for all the treatment types, ranging from 69% ± 26% to 81% ± 8% (Table 1; Figure 3c) although no significant difference was found among the five different treatment types regarding the removal of components C1 or C2 (ANOVA, *p* > 0.05; Figure 3c). Our results indicate that the protein-like fluorescence is the most biodegradable component among the three decomposed PARAFAC components irrespective of treatment type. The finding agreed well with a number of previous studies reporting effective consumption of protein-like components by bacteria [30,31,34]. The fulvic-like fluorescence (C2) was mostly depleted by the biological treatment processes for all five types, ranging from 37% ± 27% to 65% ± 35% (Figure 3c). However, there was a wide variation for A2O processes. The removal of component C2 in our study is in good agreement with a prior report, in which efficient removal of two similar components was observed for water recycling treatment plants in Australia [19]. In contrast, humic-like fluorescence (C3) appeared to have accumulated during the biological treatment as shown by the increase of the fluorescence intensity for all the wastewater treatment plants investigated (Figure 3d). However, the increase in the *F*_max_ of C3 was not statistically distinguished among the five different treatment types (ANOVA, *p* > 0.05). It was reported that a similar fluorescence component might originate from terrestrial sources, but the component is also known to be resistant to bio- and photo-degradation as well as adsorptive removal processes [4]. The microbial humification of organic matter may also explain the enrichment of the component. For example, the phenomenon was reported from laboratory incubations [31,34,35] and also from natural aquatic environments [36,37]. In addition, the accumulation of component C3 may be in part associated with the transformation of component C2 and/or the release of particulate organic matter (POM) during the wastewater treatment processes [38].

The *F*_max_ of C1, C2 and C3 in the effluents were 42 ± 18, 89 ± 30, and 16 ± 17 QSE, respectively. Due to the different treatability of the wastewater treatment plants for each fluorescence component, the relative contribution of each PARAFAC component to the total fluorescence in the effluents was not the same (Figure 3a,b). Notably, %C1 decreased from 54% ± 8% to 28% ± 8% from the influent to the effluent, while %C2 increased from 43% ± 6% to 62% ± 9%. The %C3 showed net increase from 3% ± 7% to 10% ± 8%. As a result, fulvic-like fluorescence (C2) was the most dominant component in the effluents for all the wastewater treatments plants investigated. The protein-like components are commonly correlated to the bioavailability of DOC in aquatic environments [29] Therefore, our results suggest that the PARAFAC components may be effectively used to estimate the concentration of DOM as well as its bioavailability for all the types of the wastewater treatment plants. It is notable that the concentration and the bioavailability of the effluent DOM were higher than those of natural aquatic DOM that have been more frequently explored for the estimation using EEM-PARAFAC technique [7,16]. In addition, the influents of the wastewater treatment plants in this study were municipal sewage, thus the characteristics and treatability of DOM may be different for other types of wastewater such as industrial wastewater.

### 3.4. Estimating TOC, DOC, BOD, and COD Using EEM-PARAFAC

**Figure 4.**
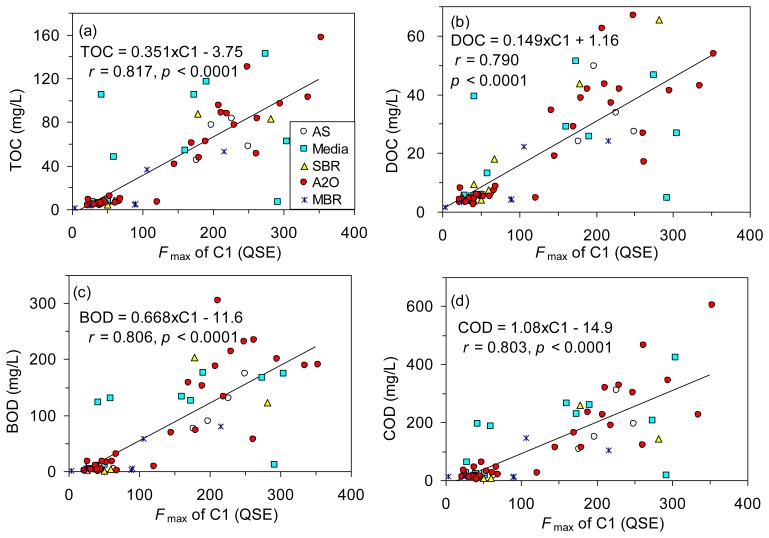
Relationships between the fluorescence intensity of C1 and (**a**) TOC, (**b**) DOC, (**c**) BOD, and (**d**) COD.

The levels of TOC, DOC, BOD and COD are all measures of the organic matter concentration in wastewater, and fluorescence spectroscopy targets only fluorescent fractions of the organic matter. Nevertheless, many studies have revealed close correlations between the traditional water quality parameters and fluorescence information from EEM-PARAFAC [5,14,16], which can lead to the potential use of rapid online monitoring at a high sensitivity. In order to test the applicability of using EEM-PARAFAC technique to estimate the organic matter concentration of wastewater in the various biological treatment plants, correlations between the conventional parameters and fluorescence intensities of the three PARAFAC components were compared in Table 2. In addition to Pearson's *r* values, Spearman *ρ* values were also calculated because most of the data appeared skewed and more distributed toward low concentration ranges [16]. All the organic matter concentrations were significantly correlated with the *F*_max_ of C1 (*r* = 0.790–0.817, *p* < 0.001; Figure 4) and C2 (*r* = 0.607–0.679, *p* < 0.001; Table 2). In contrast, the same correlations with the *F*_max_ of humic-like fluorescence (C3) were not significant based on Pearson's *r* values (*p* > 0.05) or they were weak based on Spearman *ρ* values (Table 2). The results of principal component analysis also showed that the *F*_max_ of C1 was related most strongly with TOC, DOC, BOD and COD, while the loadings for the *F*_max_ of C3 were much different from those of other indices (Figure S2). Our results were similar to those reported in studies using wastewater [5,13]. However, dissimilar results were reported for the river water where humic-like fluorescence components were dominant [16,33,39]. Our results clearly demonstrated that the bulk concentration of organic matter can be best estimated by monitoring the fluorescence intensity of protein-like fluorescence component (C1).

We further tested whether the organic matter concentration could be better described with a combination of the three PARAFAC components based on a stepwise multiple regression method. Our results showed that TOC, DOC, and COD could be sufficiently estimated by the *F*_max_ of C1 alone because the correlation coefficients were not notably improved by adding the *F*_max_ of C2 and C3 as additional independent variables. The estimation of BOD was improved to a limited extent when both the *F*_max_ of C1 and C3 were used for the estimation (BOD = 0.679 × C1 − 0.432×C3 – 6.06, *r* = 0.822), as compared with the prediction using the *F*_max_ of C1 alone (*r* = 0.806, Table 2, Figure 4). The results supported the potential of the protein-like fluorescence as a strong surrogate for biodegradable organic matter concentration as mentioned above.

Except for DOC, all other three organic matter parameters (*i.e.*, TOC, BOD and COD) are associated with both DOC and POC. For example, the contribution of DOC to TOC reached from 20% to 100% with an average value of 69% ± 22% in this study. In addition, there may be complex desorption-adsorption interactions between POC and DOC within the wastewater treatment processes. This suggests that including POC to the regression may help improve the estimation of TOC, BOD, and COD.

The inclusion of POC for this study notably enhanced the prediction capability of the *F*_max_ of C1 for BOD and COD with *r* values increased from 0.803-0.806 to 0.886-0.896 (Table 3). The strong prediction (*r* = 0.958, *p* < 0.001) for TOC using the regression based on the *F*_max_ of C1 and POC (*r* = 0.969, Table 3) was partially attributed to the fact that POC was calculated from TOC (*i.e.*, TOC minus DOC) for this study.

However, the problem in using POC for monitoring lies in relatively high cost and long time for the measurement, thus developing another surrogate to represent particulate matter in wastewater, which can be easily monitored on-site, is required. It is well known that POC is highly associated with SS in wastewater, which can be measured by in situ turbidity meters. Our result also presents the close association between the two parameters, as revealed by the high correlation coefficient (*r* = 0.799, *p* < 0.001). For this study, multiple regression analyses showed that the predictions of TOC, BOD and COD were much improved when both the *F*_max_ of C1 and SS were included in the regressions (*r* = 0.856–0.865; Table 3), as compared with those with the *F*_max_ of C1 only (*r* = 0.803–0.817; Table 2).

### 3.5. Estimating the Biodegradability of TOC Using EEM-PARAFAC

The biodegradability of organic matter is an important factor for evaluating both the treatability of organic matter in wastewater treatment plants and biogeochemical roles of effluents in the receiving water [7,9]. It is also related to various features of organic matter, such as the molecular weight [40], the N:C ratio [41] and the fraction of protein-like materials [10,29,31]. Considering that the ratio of BOD/COD has been used as a good surrogate for the biodegradability [10,11], we examined here the possibility that the BOD/COD ratio could be estimated by the chemical composition information derived from the EEM-PARAFAC, which has the potential of in situ real-time monitoring.

For this study, correlation analyses showed that BOD/COD correlated significantly (*p* < 0.001) with %C1, %C2, and %C3 (*r* = −0.568 to 0.613; *ρ* = −0.616 to 0.610), and the ratios of C2/C1, C3/C1 and C3/C2 (*r* = −0.419 to −0.598; *ρ* = −0.544 to −0.670) in this study (Table 4).

**Figure 5.**
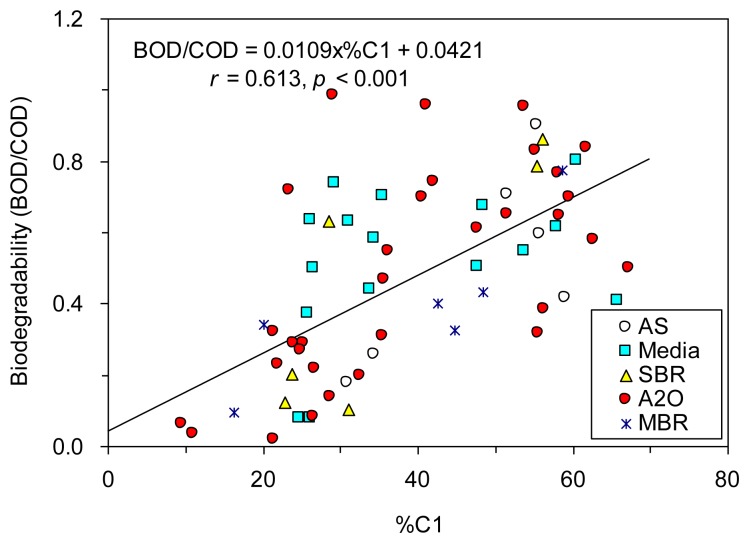
Relationship between the biodegradability of TOC (BOD/COD) and the fraction of protein-like C1 in total fluorescence (%C1).

PCA results also showed that the principal component 1 (PC1), which explained 63.0% of total variance, correlated negatively with both %C1 and BOD/COD, and positively with other indices (Figure S3). The percent of protein-like fluorescence in total fluorescence (*i.e.*, %C1) showed the strongest positive relationship with BOD/COD (Figure 5), which agreed well with the reported property of protein-like component susceptible to biodegradation [10,29–31] and also with the high removal rate observed for our biological wastewater treatment plants (Figure 3c). Although the removal of fulvic-like fluorescence was observed here as well (Figure 3c), it occurred only to a smaller extent compared to component C1. %C2 and C2/C1 were negatively correlated with BOD/COD due to the negative relationships established between %C1 and %C2 (*r* = −0.873, *p* < 0.001). %C3, C3/C1, and C3/C2 also showed negative correlations with BOD/COD probably because the humic-like fluorescence (component C3) is more stable to biodegradation [4], and it was even produced in most of the wastewater treatment plants investigated here.

**Figure 6.**
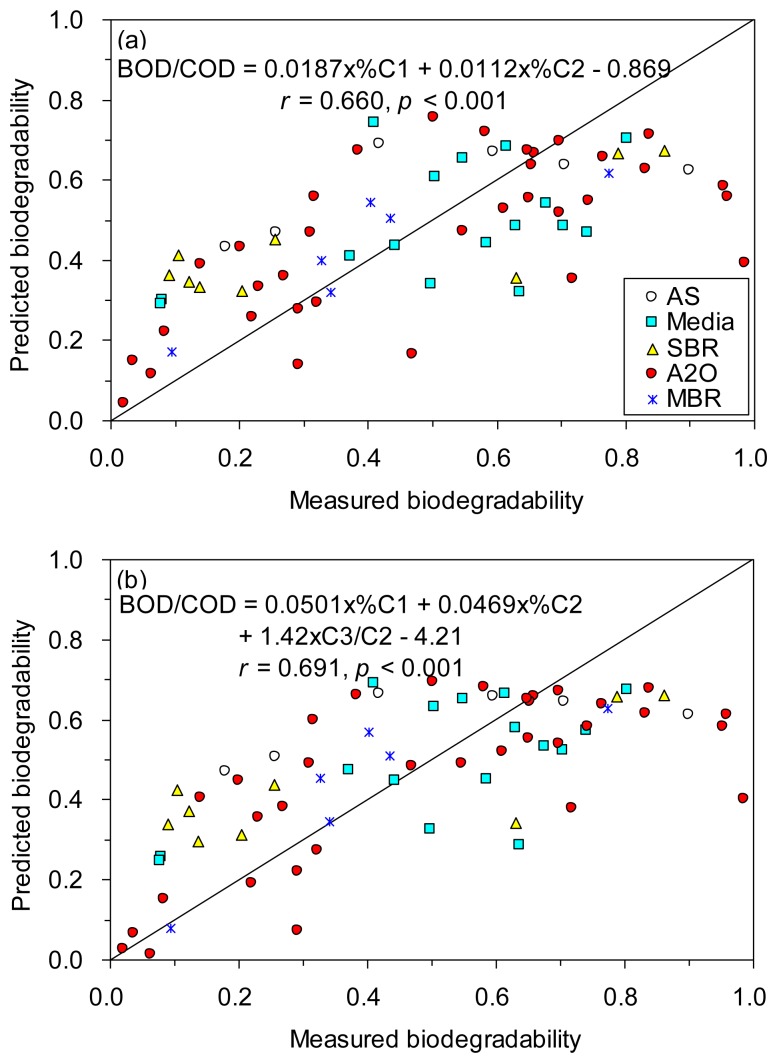
Correlations between the measured biodegradability of TOC (BOD/COD) and the predicted biodegradability by multiple regression methods based on (**a**) %C1 and %C2, and (**b**) %C1, %C2 and C3/C2.

Encouraged by the significant correlations found between the six fluorescence component proxies and BOD/COD, we tested whether the prediction of biodegradability of TOC could be enhanced by using stepwise multiple regressions. Our results showed that the prediction was improved based on %C1 and %C2 (*r* = 0.660; Figure 6a), and based on %C1, %C2 and C3/C2 (*r* = 0.691; Figure 6b), as compared to based on %C1 alone (*r* = 0.613, Figure 5).

Again, our study demonstrated that the biodegradability of organic matter in wastewater could be roughly estimated by utilizing the chemical composition proxies derived from EEM-PARAFAC, irrespective of the biological treatment types. Because the biodegradability of organic matter is likely to be in part associated with that of POC, it is expected that the prediction could be enhanced further by including the proxies for the biodegradable POC fraction in the future.

## 4. Conclusions

The majority of TOC, DOC, BOD, COD, and SS were effectively removed in the 22 studied wastewater treatment plants adopting five different types of the treatment processes. Three fluorescent components were decomposed from the fluorescence EEM data of the influent and the effluent samples, each of which responded differently to the wastewater treatment. The protein-like fluorescence (component C1) was removed the most efficiently, followed by the fulvic-like fluorescence (component C2). Humic-like fluorescence (component C3) appeared to be accumulated during the biological wastewater treatments. The relative distributions of the three fluorescence components largely changed with decreases in %C1 but increased in %C2 and %C3. The levels of TOC, DOC, BOD and COD in the wastewater could be roughly estimated by using the fluorescence intensity of C1. The prediction capability was improved by using multiple regressions based on the *F*_max_ of C1 and SS, both of which are possibly monitored on-site. The biodegradability of TOC was well correlated with six chemical composition parameters derived from EEM-PARAFAC with the highest correlation coefficient shown for %C1. The prediction efficacy of the biodegradability was further enhanced using multiple regressions based on %C1, %C2 and C3/C2. Overall, our study demonstrated that EEM-PARAFAC technique could be effectively used for on-site monitoring of the concentration, chemical composition and biodegradability of organic matter in many wastewater treatment plants.

## Supplementary Material



## Figures and Tables

**Table 1.** Summary of BOD, COD, TOC, DOC, SS, and fluorescence intensities of C1, C2, and C3 in the influents and effluents, and the relative changes (%) in the five types of treatment plants.

**Treatment**	**BOD** **(mg/L)**	**COD** **(mg/L)**	**TOC** **(mg/L)**	**DOC** **(mg/L)**	**SS** **(mg/L)**	***F*_max_** **of C1** **(QSE)**	***F*_max_** **of C2** **(QSE)**	***F*_max_** **of C3^a^** **(QSE)**

Influent								

AS (*n* = 2)	76–90 b (83 ± 9) c	108–151 (129 ± 30)	45–77 (61 ± 23)	24–50 (37 ± 18)	34–40 (37 ± 4)	177–197 (187 ± 14)	157–167 (162 ± 7)	0
Media (*n* = 5)	125–175 (155 ± 24)	207–423 (277 ± 85)	54–142 (96 ± 37)	26–51 (36 ± 12)	60–283 (129 ± 97)	161–305 (221 ± 65)	150–180 (169 ± 13)	0–26 (5.2 ± 12)
SBR (*n* = 3)	11–204 (113 ± 97)	43–259 (149 ± 108)	19–88 (63 ± 39)	18–66 (43 ± 24)	16–110 (64 ± 47)	67–282 (176 ± 107)	82–220 (149 ± 69)	0–15 (5.1 ± 8.9)
A2O (*n* = 10)	57–234 (143 ± 61)	112–467 (223 ± 111)	41–103 (74 ± 22)	17–62 (36 ± 13)	38–210 (95 ± 51)	142–335 (224 ± 64)	115–240 (162 ± 40)	0–22 (4.3 ± 7.9)
MBR (*n* = 2)	59–80 (70 ± 15)	104–147 (125 ± 31)	37–53 (45 ± 11)	22–24 (23 ± 1)	12–68 (40 ± 40)	106–216 (161 ± 78)	128–137 (133 ± 6)	5.5–24 (15 ± 13)
All (*n* = 22)	11–234 (129 ± 59)	43–467 (208 ± 104)	19–142 (74 ± 30)	17–66 (36 ± 14)	12–283 (88 ± 64)	67–335 (208 ±67)	82–240 (159 ± 36)	0–26 (5.2 ± 9.0)

Effluent								

AS (*n* = 2)	3.2–4.6 (3.9 ± 1.0)	18–18 (18 ± 0)	4.4–7.7 (6.0 ± 2.3)	3.8–6.4 (5.1 ± 1.9)	0.2–0.6 (0.4 ± 0.3)	37–64 (51 ± 19)	78–117 (97 ± 27)	5.3–6.4 (5.8 ± 0.8)
Media (*n* = 5)	2.0–12 (7.1 ± 3.9)	12–26 (18 ± 7)	4.4–8.0 (5.7 ± 1.3)	3.7–5.9 (4.7 ± 0.9)	0.8–3.3 (1.9 ± 1.1)	27–52 (37 ± 9)	67–124 (88 ± 24)	0–19 (8.7 ± 7.7)
SBR (*n* = 3)	0.8–2.4 (1.5 ± 0.9)	8.0–20 (12 ± 7)	4.4–5.1 (4.6 ± 0.4)	4.0–4.9 (4.4 ± 0.5)	0.8–3.8 (2.0 ± 1.6)	26–50 (39 ± 12)	74–102 (85 ± 15)	7.6–17 (12 ± 5)
A2O (*n* = 10)	0.2–18 (5.8 ± 5.6)	4.0–46 (18 ± 12)	3.5–12 (5.9 ± 2.8)	2.6–8.6 (4.5 ± 1.7)	0.2–5.6 (2.6 ± 1.9)	22–69 (42 ± 15)	50–151 (97 ± 32)	2.3–65 (22 ± 22)
MBR (*n* = 2)	1.3–3.3 (2.3 ± 1.4)	10–14 (12 ± 3)	1.7–4.7 (3.2 ± 2.1)	1.6–4.3 (2.9 ± 1.9)	0.4–1.2 (0.8 ± 0.6)	3–88 (46 ± 60)	14–76 (45 ± 44)	3.9–33 (18 ± 21)
All (*n* = 22)	0.2–18 (5.0 ± 5.1)	4.0–46 (17 ± 9)	1.7–12 (5.5 ± 2.2)	1.6–8.6 (4.4 ± 1.4)	0.2–5.6 (2.0 ± 1.6)	3–88 (42 ± 18)	14–151 (89 ± 30)	0–65 (16 ± 17)

Removal efficiency (%) or percent increase in the *F*_max_ of C3 (QSE)								

AS (*n* = 2)	94–96 (95 ± 2)	83–88 (86 ± 3)	83–94 (89 ± 8)	73–92 (83 ± 13)	98–100 (99 ± 1)	64–81 (72 ± 12)	30–51 (40 ± 14)	5.3–6.4 (5.8 ± 0.8)
Media (*n* = 5)	93–99 (95 ± 3)	91–95 (94 ± 2)	89–96 (93 ± 3)	77–93 (85 ± 6)	96–100 (98 ± 2)	73–91 (81 ± 8)	30–57 (48 ± 11)	−7–14 (3.5 ± 7.8)
SBR (*n* = 3)	90–99 (96 ± 5)	81–94 (89 ± 7)	76–95 (88 ± 10)	78–94 (87 ± 8)	76–99 (91 ± 13)	40–86 (69 ± 26)	11–54 (37 ± 23)	1.8–10 (6.6 ± 4.4)
A2O (*n* = 10)	89–100 (97 ± 4)	82–98 (91 ± 5)	77–96 (91 ± 7)	54–95 (85 ± 12)	87–100 (96 ± 4)	53–88 (80 ± 11)	−20–62 (37 ± 27)	−2.0–31 (13 ± 13)
MBR (*n* = 2)	96–98 (97 ± 1)	90–90 (90 ± 0)	91–95 (93 ± 3)	82–93 (88 ± 7)	97–98 (97 ± 1)	59–97 (78 ± 27)	40–90 (65 ± 35)	−1.7–9.2 (3.8 ± 7.7)
All (*n* = 22)	89–100 (96 ± 3)	81–98 (91 ± 4)	76–96 (91 ± 6)	54–95 (85 ± 10)	76–100 (96 ± 5)	40–97 (78 ± 14)	−20–90 (42 ± 23)	−7–31 (8.2 ± 9.9)

a An exceptionally high *F*_max_ value of C3 in the influent (231 QSE) was excluded;

b Range in the values;

c Mean ± standard deviation.

**Table 2.** Correlation coefficients (Pearson *r* values and Spearman *ρ* values) between conventional organic matter concentration indices and fluorescence intensities of three PARAFAC components (n = 71).

	**TOC**	**DOC**	**BOD**	**COD**
*F*_max_ of C1	0.817 ***, a	0.790 ***	0.806 ***	0.803 ***
	0.812 ***, b	0.787 ***	0.801 ***	0.719 ***
*F*_max_ of C2	0.665 ***	0.679 ***	0.644 ***	0.607 ***
	0.750 ***	0.754 ***	0.652 ***	0.635 ***
*F*_max_ of C3	−0.025	−0.037	−0.093	−0.018
	−0.254 *	−0.233	−0.360 **	−0.303 *

*** *p* < 0.001;

** *p* < 0.01;

* *p* < 0.05;

a Pearson *r* values;

b Spearman *ρ* values.

**Table 3.** Prediction of water quality parameters using multiple regressions based on *F*_max_ of C1 and POC or *F*_max_ of C1 and SS.

	**Regression Equation**	**Correlation Coefficient (*r*)**
TOC	TOC = 0.0947 × C1 + 1.27 × POC + 2.49 a	0.969
	TOC = 0.192 × C1 + 0.292 × SS + 1.01 b	0.865
BOD	BOD = 0.318 × C1 + 1.74 × POC – 3.12	0.886
	BOD = 0.412 × C1 + 0.544 × SS – 5.92	0.856
COD	COD = 0.470 × C1 + 3.03 × POC – 0.052	0.896
	COD = 0.537 × C1 + 0.909 × SS + 3.62	0.860

a n = 71;

b n = 70; Data with an exceptionally high SS concentration (550 mg·L^−1^) was excluded from the regressions.

**Table 4.** Correlation coefficients (Pearson *r* values and Spearman *ρ* values) between biodegradability (BOD/COD) and chemical composition proxies based on EEM-PARAFAC (n =71, *p* < 0.001).

	**%C1**	**%C2**	**%C3**	**C2/C1**	**C3/C1**	**C3/C2**
**Pearson** ***r*** **values**	0.613	−0.415	−0.568	−0.544	−0.598	−0.419
**Spearman** ***ρ*** **values**	0.610	−0.422	−0.616	−0.553	−0.670	−0.544
