# Supplementary material for: Estimating the Concentration and Biodegradability of Organic Matter in 22 Wastewater Treatment Plants Using Fluorescence Excitation Emission Matrices and Parallel Factor Analysis
Source: Sensors (Basel). 2014 Jan 20;14(1):1771–86. doi: 10.3390/s140101771 (PMC3926638; doi:10.3390/s140101771)
Supplement: Supplementary file 1 [file sensors-14-01771-s002.pdf]

OPEN ACCESS

sensors

ISSN 1424-8220

www.mdpi.com/journal/sensors

Supplementary Information

Estimating the Concentration and Biodegradability of Organic Matter in 22 Wastewater Treatment Plants Using Fluorescence Excitation Emission Matrices and Parallel Factor Analysis. *Sensors* 2014, *14*, 1771-1786

Liyang Yang [1], Hyun-Sang Shin [2] and Jin Hur [1,*]

[1] Department of Environment & Energy, Sejong University, 98 Gunja-dong, Gwangjin-ku, Seoul 143-747, Korea; E-Mail: yangliyang2002@163.com

[2] Department of Environmental Engineering, Seoul National University of Science and Technology, Seoul 139-743, Korea; E-Mail: hyuns@seoultech.ac.kr

* Author to whom correspondence should be addressed; E-Mail: jinhur@sejong.edu; Tel.: +82-2-3408-3826; Fax: +82-2-3408-4320.

Table S1. Summary of wastewater treatment plants.

WWTP name	Longitude (°)	Latitude (°)	Treatment Capacity (m^3/day)	Biological Treatment Types
Tancheon (TC)	127.09	37.50	1,100,000	AS
Nanji (NJ)	126.85	37.59	1,000,000	AS
Pangyo (PG)	127.10	37.40	47,000	Media
Wonju (WJ)	127.94	37.39	130,000	Media
Jecheon (JC)	128.19	37.13	70,000	Media
Janggye (JG)	127.57	35.74	2,000	Media
Jinju (JJ)	128.13	35.21	150,000	Media
Wonning (WN)	128.79	35.62	80,000	SBR
Sintaein (ST)	126.87	35.69	2,600	SBR
Gangchon (GC)	127.64	37.81	4,000	SBR
Gwangju (GJ)	127.28	37.39	25,000	A2O
Yangpyeong (YP)	127.45	37.51	16,000	A2O
Jungnang (JR)	127.06	37.56	1,710,000	A2O
Geumchon (KC)	126.77	37.76	27,000	A2O
Gapyeong (KP)	127.52	37.82	11,500	A2O
Jincheon (JC)	127.50	36.84	8,000	A2O
Gumi (GM)	128.40	36.09	330,000	A2O
Iksan (IS)	126.98	35.93	100,000	A2O
Damyang (DY)	126.96	35.31	7,000	A2O
Naesu (NS)	127.52	36.73	8,000	A2O
Daesin (DS)	127.56	37.38	800	MBR
Munsan (MS)	126.76	37.86	9,500	MBR

Figure S1. Locations of wastewater treatment plants in this study.

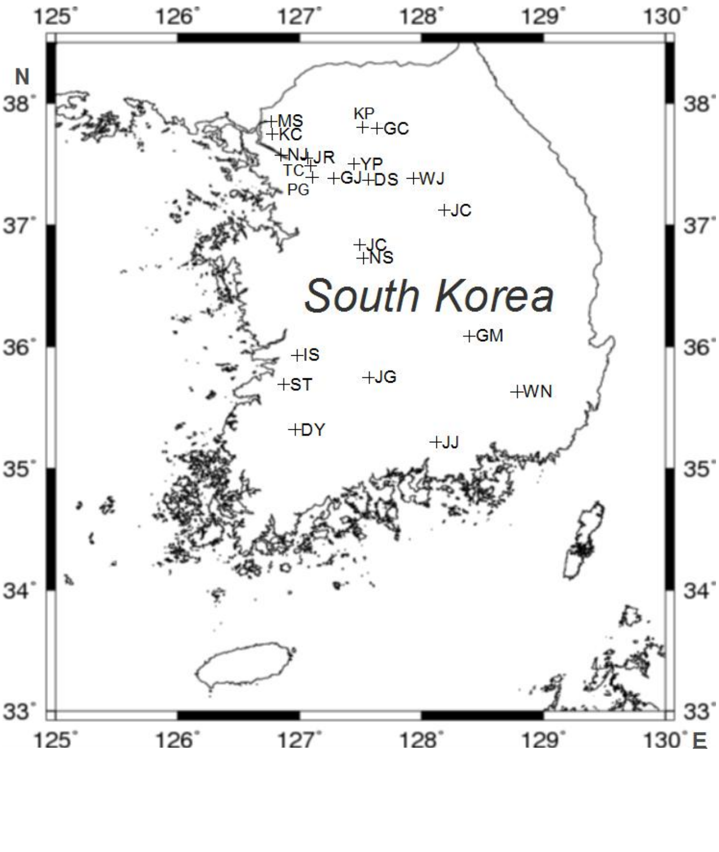

Figure S2. Results of principle component analysis (PCA) based on fluorescence intensities of C1, C2, and C3, TOC, DOC, BOD and COD.

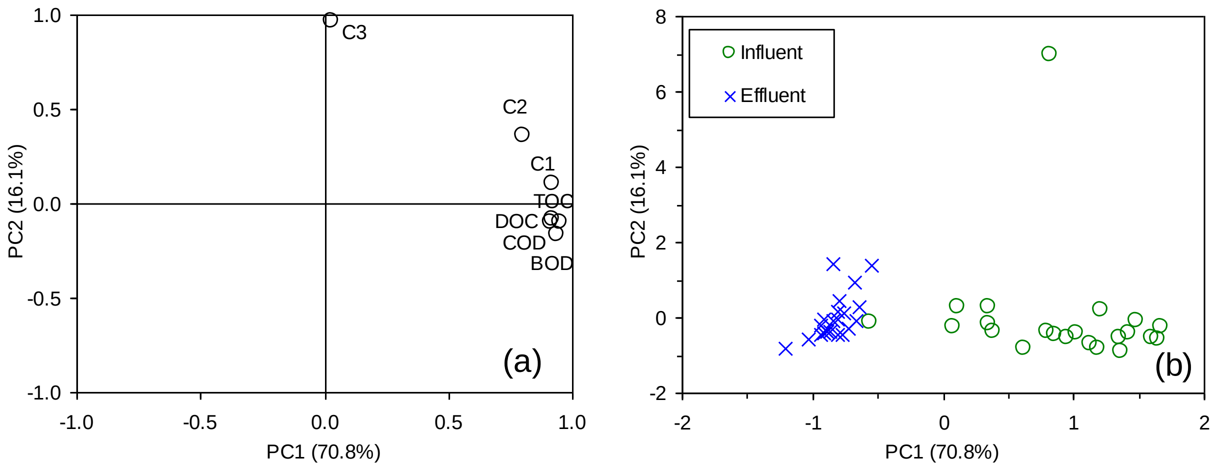

Figure S3. Results of principle component analysis (PCA) based on %C1, %C2, %C3, C2/C1, C3/C1, C3/C2, and BOD/COD.

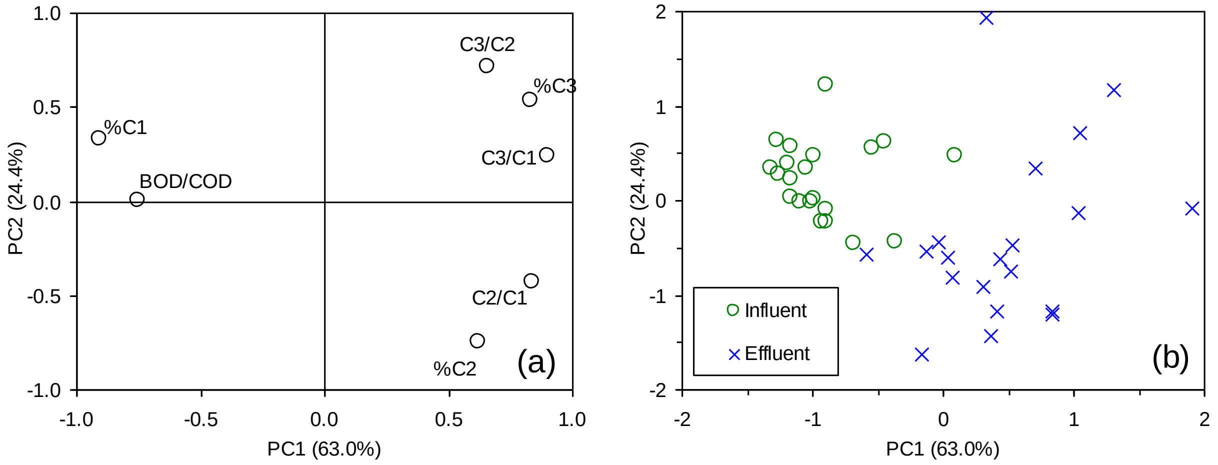

Table S2. Detailed monitoring data for the 22 wastewater treatment plants.

WWTP Name	Sample Type	BOD (mg/L)	COD (mg/L)	TOC (mg/L)	DOC (mg/L)	POC (mg/L)	SS (g/L)	BOD/COD	F_{max} of C 1 (QSE)	F_{max} of C 2 (QSE)	F_{max} of C 3 (QSE)	%C1 (%)	%C2 (%)	%C3 (%)	C2/C1	C3/C1	C3/C2
**AS**																	
TC	Influent	89.6	150.6	77.5	49.6	27.8	40.0	0.60	197.2	157.2	0.0	55.6	44.4	0.0	0.80	0.00	0.00
TC	Before P removal	130.2	311.0	83.3	33.7	49.6	193.3	0.42	226.6	156.1	1.5	59.0	40.6	0.4	0.69	0.01	0.01
TC	Effluent	3.2	18.0	4.4	3.8	0.6	0.2	0.18	37.1	77.8	5.3	30.9	64.7	4.4	2.10	0.14	0.07
NJ	Influent	76.3	108.0	45.1	24.1	21.0	34.0	0.71	177.1	166.7	0.0	51.5	48.5	0.0	0.94	0.00	0.00
NJ	Before P removal	175.0	194.4	58.1	27.4	30.6	150.0	0.90	249.8	183.8	17.2	55.4	40.8	3.8	0.74	0.07	0.09
NJ	Effluent	4.6	18.0	7.7	6.4	1.3	0.6	0.26	64.2	116.6	6.4	34.3	62.3	3.4	1.82	0.10	0.05
**Media**																	
PG	Influent	166.6	207.4	142.3	46.6	95.7	166.0	0.80	274.7	179.9	0.0	60.4	39.6	0.0	0.65	0.00	0.00
PG	Before P removal	130.9	185.8	47.8	13.1	34.7	23.4	0.70	59.1	102.6	5.0	35.5	61.5	3.0	1.74	0.08	0.05
PG	Effluent	4.3	11.6	5.5	4.8	0.7	3.3	0.37	31.0	85.1	3.9	25.8	70.9	3.3	2.75	0.13	0.05
WJ	Influent	175.4	259.2	116.8	25.6	91.3	70.0	0.68	190.7	177.5	26.0	48.4	45.0	6.6	0.93	0.14	0.15
WJ	Before P removal	11.0	17.3	7.1	5.2	1.9	4.8	0.64	51.6	122.9	22.6	26.2	62.4	11.5	2.38	0.44	0.18
WJ	Effluent	12.0	24.0	8.0	5.9	2.1	2.0	0.50	51.5	124.4	19.0	26.4	63.8	9.7	2.42	0.37	0.15
JC	Influent	125.5	229.0	104.7	51.4	53.3	65.0	0.55	173.6	150.2	0.0	53.6	46.4	0.0	0.87	0.00	0.00
JC	Before P removal	9.7	22.0	5.8	3.5	2.3	2.4	0.44	40.0	71.7	7.1	33.7	60.3	6.0	1.79	0.18	0.10
JC	Effluent	8.2	14.0	5.0	3.7	1.3	1.0	0.59	38.5	66.8	6.7	34.4	59.7	6.0	1.74	0.17	0.10
JG	Influent	173.6	423.4	62.4	26.7	35.7	283.3	0.41	304.9	158.9	0.0	65.7	34.3	0.0	0.52	0.00	0.00
JG	Before P removal	5.0	62.0	7.1	5.5	1.6	2.8	0.08	28.6	66.6	14.3	26.2	60.8	13.1	2.32	0.50	0.21
JG	Effluent	2.0	26.0	4.4	4.0	0.5	0.8	0.08	26.7	67.5	14.0	24.7	62.4	12.9	2.53	0.53	0.21
JJ	Influent	133.7	264.8	53.7	29.1	24.6	60.0	0.50	160.6	176.7	0.0	47.6	52.4	0.0	1.10	0.00	0.00
JJ	Before P removal	122.5	194.4	105.2	39.3	65.9	230.0	0.63	42.0	93.2	0.0	31.1	68.9	0.0	2.22	0.00	0.00
JJ	Before P removal	11.1	18.0	6.4	4.6	1.8	4.0	0.61	292.2	213.1	0.0	57.8	42.2	0.0	0.73	0.00	0.00
JJ	Effluent	8.9	12.0	5.8	5.3	0.6	2.6	0.74	38.8	94.1	0.0	29.2	70.8	0.0	2.43	0.00	0.00
**SBR**																	
WN	Influent	204.1	259.2	87.7	43.9	43.8	110.0	0.79	178.1	143.9	0.0	55.3	44.7	0.0	0.81	0.00	0.00
WN	Before P removal	7.4	36.0	6.1	5.4	0.7	6.4	0.20	24.1	68.3	9.6	23.7	66.9	9.4	2.83	0.40	0.14
WN	Effluent	2.4	20.0	5.1	4.9	0.2	1.4	0.12	25.5	78.4	7.6	22.9	70.3	6.8	3.07	0.30	0.10
ST	Influent	123.2	143.2	83.6	65.6	18.0	66.0	0.86	281.8	220.4	0.0	56.1	43.9	0.0	0.78	0.00	0.00
ST	Before P removal	5.0	8.0	8.4	7.5	0.9	2.8	0.63	59.7	130.3	19.9	28.4	62.1	9.5	2.18	0.33	0.15
ST	Effluent	0.8	8.0	4.4	4.2	0.2	0.8	0.11	50.3	101.8	10.5	31.0	62.6	6.5	2.02	0.21	0.10
GC	Influent	11.0	43.2	18.7	18.0	0.7	16.0	0.25	66.9	82.4	15.4	40.6	50.0	9.3	1.23	0.23	0.19
GC	Before P removal	1.3	14.0	10.4	9.5	0.9	12.8	0.09	40.6	77.1	13.9	30.8	58.6	10.6	1.90	0.34	0.18
GC	Effluent	1.1	8.0	4.4	4.0	0.4	3.8	0.14	40.3	73.6	17.2	30.8	56.1	13.1	1.83	0.43	0.23

Table S2. *Cont.*

WWTP Name	Sample Type	BOD (mg/L)	COD (mg/L)	TOC (mg/L)	DOC (mg/L)	POC (mg/L)	SS (g/L)	BOD/COD	F_{max} of C 1 (QSE)	F_{max} of C 2 (QSE)	F_{max} of C 3 (QSE)	%C1 (%)	%C2 (%)	%C3 (%)	C2/C1	C3/C1	C3/C2
**A2O**																	
GJ	Influent	133.7	203.0	73.0	34.7	38.3	116.2	0.66	142.1	114.9	0.0	55.3	44.7	0.0	0.81	0.00	0.00
GJ	Before P removal	17.7	18.0	5.6	4.2	1.4	6.4	0.99	26.3	57.7	6.3	29.1	63.9	7.0	2.20	0.24	0.11
GJ	Effluent	1.7	12.0	4.0	3.3	0.8	1.4	0.14	22.1	50.1	5.2	28.6	64.7	6.7	2.26	0.23	0.10
YP	Influent	132.7	190.1	87.8	37.0	50.8	86.0	0.70	219.3	149.1	0.0	59.5	40.5	0.0	0.68	0.00	0.00
YP	Before P removal	3.2	16.0	4.4	4.2	0.2	2.4	0.20	40.3	76.8	7.0	32.5	61.9	5.7	1.91	0.17	0.09
YP	Effluent	4.4	14.0	3.5	3.2	0.4	2.0	0.31	39.1	66.5	4.8	35.4	60.2	4.4	1.70	0.12	0.07
JR	Influent	152.4	233.3	62.6	41.7	20.9	38.0	0.65	189.1	178.7	0.0	51.4	48.6	0.0	0.94	0.00	0.00
JR	Influent	304.9	319.7	88.6	43.5	45.1	114.0	0.95	211.5	158.8	23.8	53.7	40.3	6.0	0.75	0.11	0.15
JR	Before P removal	9.2	24.0	6.5	4.7	1.8	3.2	0.38	121.4	94.1	0.0	56.3	43.7	0.0	0.77	0.00	0.00
JR	Effluent	16.4	30.0	12.1	5.2	6.9	4.8	0.55	53.8	88.5	6.8	36.1	59.4	4.5	1.64	0.13	0.08
KC	Influent	200.9	345.6	97.1	41.3	55.9	90.0	0.58	295.2	176.4	0.0	62.6	37.4	0.0	0.60	0.00	0.00
KC	Before P removal	30.7	44.0	7.3	7.2	0.0	4.0	0.70	67.5	93.7	5.4	40.5	56.2	3.2	1.39	0.08	0.06
KC	Effluent	17.9	24.0	5.7	5.2	0.5	5.2	0.74	61.7	83.2	2.3	42.0	56.5	1.5	1.35	0.04	0.03
KP	Influent	233.8	466.6	83.3	16.9	66.4	120.0	0.50	262.7	128.7	0.0	67.1	32.9	0.0	0.49	0.00	0.00
KP	Influent	213.5	328.3	77.2	41.9	35.3	65.0	0.65	230.1	184.2	32.6	51.5	41.2	7.3	0.80	0.14	0.18
KP	Before P removal	17.9	61.5	6.2	5.5	0.7	1.2	0.29	48.1	114.7	27.9	25.2	60.2	14.6	2.39	0.58	0.24
KP	Effluent	10.1	45.9	5.8	4.7	1.1	1.6	0.22	38.2	79.9	24.9	26.7	55.9	17.4	2.09	0.65	0.31
JC	Influent	186.9	224.6	95.1	62.4	32.7	98.0	0.83	208.1	156.6	12.3	55.2	41.5	3.3	0.75	0.06	0.08
JC	Influent	231.7	302.4	130.8	66.9	63.9	152.0	0.77	248.4	167.7	11.7	58.1	39.2	2.7	0.68	0.05	0.07
JC	Before P removal	4.5	14.0	5.6	4.4	1.2	2.8	0.32	35.0	111.7	17.4	21.3	68.1	10.6	3.19	0.50	0.16
JC	Effluent	2.3	10.0	4.3	3.4	0.9	1.6	0.23	31.2	100.9	10.3	21.9	70.9	7.2	3.24	0.33	0.10
GM	Influent	56.7	121.0	50.9	26.8	24.1	50.0	0.47	261.3	239.7	230.8	35.7	32.8	31.5	0.92	0.88	0.96
GM	Before P removal	3.5	12.0	6.2	5.7	0.5	1.6	0.29	46.7	97.5	51.1	23.9	49.9	26.2	2.09	1.10	0.52
GM	Effluent	0.2	10.0	4.5	4.4	0.2	0.2	0.02	42.0	90.4	64.8	21.3	45.8	32.9	2.15	1.54	0.72
IS	Influent	68.6	112.3	41.2	18.8	22.3	102.0	0.61	145.7	137.5	22.2	47.7	45.0	7.3	0.94	0.15	0.16
IS	Influent	191.1	604.8	157.7	53.9	103.7	550.0	0.32	353.4	220.8	62.6	55.5	34.7	9.8	0.62	0.18	0.28
IS	Effluent	1.7	20.0	9.6	8.6	1.0	5.6	0.08	68.9	138.5	53.2	26.4	53.1	20.4	2.01	0.77	0.38
DY	Influent	72.8	112.3	47.0	38.9	8.1	38.0	0.65	180.4	125.7	3.9	58.2	40.6	1.3	0.70	0.02	0.03
DY	Before P removal	2.2	34.0	8.7	8.1	0.5	3.2	0.06	23.4	179.0	45.3	9.4	72.3	18.3	7.65	1.94	0.25
DY	Effluent	0.5	14.0	4.3	4.2	0.1	1.3	0.04	22.9	151.3	34.4	11.0	72.5	16.5	6.62	1.50	0.23
NS	Influent	188.3	224.6	102.7	43.0	59.6	210.0	0.84	335.2	208.1	0.0	61.7	38.3	0.0	0.62	0.00	0.00

Table S2. *Cont.*

WWTP Name	Sample Type	BOD (mg/L)	COD (mg/L)	TOC (mg/L)	DOC (mg/L)	POC (mg/L)	SS (g/L)	BOD/COD	F_{max} of C 1 (QSE)	F_{max} of C 2 (QSE)	F_{max} of C 3 (QSE)	%C1 (%)	%C2 (%)	%C3 (%)	C2/C1	C3/C1	C3/C2
NS	Influent	157.5	164.2	60.4	29.1	31.4	116.0	0.96	170.1	244.2	0.0	41.1	58.9	0.0	1.44	0.00	0.00
NS	Before P removal	3.8	14.0	7.7	5.9	1.8	7.0	0.27	45.2	123.9	12.4	24.9	68.3	6.8	2.74	0.27	0.10
NS	Effluent	2.9	4.0	5.7	2.6	3.1	1.8	0.72	40.7	122.5	11.5	23.3	70.1	6.6	3.01	0.28	0.09
**MBR**																	
DS	Influent	59.2	146.9	37.1	22.3	14.8	12.0	0.40	105.9	137.1	5.5	42.6	55.2	2.2	1.30	0.05	0.04
DS	Before P removal	5.5	16.0	3.5	3.4	0.1	4.4	0.34	24.0	87.2	8.7	20.0	72.7	7.3	3.63	0.36	0.10
DS	Effluent	1.3	14.0	1.7	1.6	0.1	0.4	0.09	3.5	14.2	3.9	16.2	65.8	18.0	4.05	1.11	0.27
MS	Influent	80.2	103.7	53.3	24.2	29.2	68.0	0.77	215.6	128.4	23.9	58.6	34.9	6.5	0.60	0.11	0.19
MS	Before P removal	6.1	14.0	5.2	4.5	0.8	2.8	0.44	89.5	77.0	18.2	48.5	41.7	9.8	0.86	0.20	0.24
MS	Effluent	3.3	10.0	4.7	4.3	0.5	1.2	0.33	88.5	76.4	33.1	44.7	38.6	16.7	1.16	0.37	0.43

© 2014 by the authors; licensee MDPI, Basel, Switzerland. This article is an open access article distributed under the terms and conditions of the Creative Commons Attribution license (http://creativecommons.org/licenses/by/3.0/).
